# Translating and Evaluating a Physical Activity Program for Aboriginal Elders on Noongar Boodjar (Country) — A Longitudinal Study

**DOI:** 10.3389/fpubh.2022.904158

**Published:** 2022-07-22

**Authors:** Margaret J. R. Gidgup, Marion Kickett, Angela Jacques, Tammy Weselman, Keith D. Hill, Julieann Coombes, Rebecca Ivers, Nicole Bowser, Vilma Palacios, Anne-Marie Hill

**Affiliations:** ^1^Curtin School of Allied Health, Curtin University, Perth, WA, Australia; ^2^Independent Researcher, York, WA, Australia; ^3^Institute for Health Research, The University of Notre Dame Australia, Fremantle, WA, Australia; ^4^School of Allied Health, WA Centre for Health and Ageing, The University of Western Australia, Perth, WA, Australia; ^5^Rehabilitation Ageing and Independent Living (RAIL) Research Centre, School of Primary and Allied Health Care, Monash University, Melbourne, VIC, Australia; ^6^Aboriginal and Torres Strait Islander Health Program, The George Institute for Global Health, Sydney, NSW, Australia; ^7^School of Population Health, University of New South Wales, Sydney, NSW, Australia; ^8^South West Aboriginal Medical Service Aboriginal Corporation, Bunbury, WA, Australia; ^9^North Metropolitan Public Health Unit, WA North Metropolitan Health Service, Perth, WA, Australia

**Keywords:** Aboriginal and Torres Strait Islander, aged, physical activity, First Nations, Indigenous, Elder, evaluations

## Abstract

**Objective:**

The primary aim of the study was to translate and evaluate the impact of a Physical Activity (PA) program on the physical function of older Aboriginal Elders on Noongar Boodjar (Country).

**Methods:**

A longitudinal design framed within an Indigenous methodology. Two groups, one metropolitan and one regional, of Aboriginal Elders, aged ≥45 years, participated in the Ironbark PA program. This comprised weekly strength and balance exercises followed by yarning circles. Physical function (primary outcome) and functional ability, cardiovascular risk factors (weight, waist circumference), falls efficacy and health-related quality of life were measured at baseline 6, 12 and 24 months. Data were analyzed using generalized linear mixed effects modeling.

**Results:**

Fifty-two Elders initially enrolled and of those, *n* = 23 (44.2%) Elders participated regularly for 24 months. There was a 6-month gap in program delivery due to the COVID-19 pandemic. Participants made significant improvement in physical function at 12 months compared to baseline: [short physical performance battery (SPPB) at baseline, 8.85 points (95% CI 8.10, 9.61); 12 months 10.28 (95% CI 9.44, 11.13), *p* = 0.001: gait speed at baseline 0.81 ms^−1^ (95% CI 0.60, 0.93); 12 months 1.14 (95% CI 1.01, 1.27), *p* < 0.001]. Some sustained improvement compared to baseline was still evident at 24 months after the 6-month gap in attendance [SPPB 9.60 (8.59, 10.60) *p* = 0.14, gait speed 1.11 (0.95, 1.26) *p* < 0.001]. Cardiovascular risk factors showed a non-significant improvement at 12 and 24 months compared to baseline. All participants reported that they enjoyed the program, found it culturally appropriate and would recommend it to others.

**Conclusion:**

Older Aboriginal people showed sustained improvements in physical function after engaging in a culturally appropriate PA program. Culturally appropriate PA programs provide safety, security and choice for older Aboriginal people to engage in evidence-based PA.

## Introduction

Indigenous peoples worldwide have suffered through invasion and the impacts of colonization (1, 2). It has resulted in the loss of languages, customs, and the freedom to hunt and gather on their traditional lands leading to changes in dietary intake and a sedentary lifestyle (3, 4). These consequences of colonization have negatively impacted on the health status of Indigenous peoples (3), causing disparities between the Aboriginal and non-Aboriginal people and communities in Australia. This disparity must be addressed by hearing Indigenous voices and privileging Indigenous ways of working, rather than providing only Westernized health services (2, 4).

Physical activity (PA) guidelines recommend that older adults should be participating in at least 150 min of moderate-intensity physical activity per week (5). Physical activity improves older adults' strength, balance, functional ability, mental health, cardiovascular health and reduces the risk of falls and functional decline (5, 6). However, less than half of older adults in Australia, including 27% of Aboriginal and Torres Strait Islander older adults, meet PA guidelines (7, 8). Aboriginal and Torres Strait Islander older adults are disproportionately affected by physical disability, therefore may find it difficult to participate in moderate or high intensity levels of PA (9).

However, there have been few studies worldwide that have evaluated PA programs for older Indigenous peoples (10–14). While there are a broad range of PA programs designed or recommended for older populations (15, 16), very few programs are appropriately designed to attract and engage older Indigenous peoples (17–20). A recent systematic review that included 23 studies from four countries found that acknowledging social determinants of health, cultural safety and security and taking a decolonizing approach was needed to encourage older Indigenous peoples' leadership and participation in PA programs (21). However, a review of physical activity programs focused targeting Aboriginal and Torres Strait Islander people in Australia found only 24 programs that operated in WA, and of these only one was specifically designed for older Aboriginal and Torres Strait Islander people (22). Programs that are not designed with these considerations are unlikely to succeed (21).

Taking a decolonizing approach is to “unpack” or “undo” the privilege and power that “whiteness” has always dominated with over other cultures that are different to theirs (23). It is also about trust, cultural competencies, respectfulness, recognition and acknowledgment of diversity among Aboriginal Elders, protecting Aboriginal Elders' knowledge and information shared, importance of relationships and how that contributes to a whole of community happiness and good health (24). Coombes et al. (25) describes that using an Indigenous methodological approach can ensure that the First Nation people and communities' voices are heard (25). The Ironbark PA program followed this approach as it was designed by Aboriginal researchers partnering with Elders and their local community's leadership (10). The program sought to facilitate the engagement of older Aboriginal people in PA through exercise within a culturally secure setting. These settings provided a regular venue and ongoing weekly support for the group as part of their support for the local community. Elders made physical improvements from attending the program and the community response was highly positive (10). However, there were no similar programs provided for older Aboriginal people in WA. While the Ironbark PA program fitted the needs of the Aboriginal and Torres Strait Islander communities in NSW, the “one size fits all” (17), cannot be applied to geographically and culturally disparate Aboriginal and Torres Strait Islander communities. Cultural differences may create barriers that prevent successful engagement and sustainability (18, 21). Therefore, to evaluate the Ironbark PA program in WA the program needed to be delivered with local protocols that were guided by leadership of local Aboriginal Elders. The first adaptation for undertaking the program in WA was time. The Ironbark program in NSW ran for 6 months, but in WA it was considered important to operate the program for a longer period, so that Aboriginal Elders could have time to build confidence and trust to engage in the program. This would allow the program to be evaluated for its impact over a sustained time. Therefore, the researchers aimed to operate the program for at least 18 months.

The objective of the study was to evaluate the impact of a PA program (the Ironbark program) on physical function of Aboriginal Elders on Noongar Country in WA. Secondary aims were to evaluate the impact of the program on functional mobility, cardiovascular risk factors (weight, waist circumference), falls self-efficacy and health related quality of life. Participants' feedback about the program was also sought to understand whether Aboriginal Elders enjoyed the program and felt it could be translated widely among older Aboriginal peoples in WA. This study was part of a larger project that sought to understand how the Ironbark program could be translated into WA.

## Methods

### Design

The study used a longitudinal design framed within an Indigenous methodology. This study formed part of a larger project which aimed to understand how to translate the Ironbark program into WA. Some findings of this larger project have been published previously (21, 26). The methodology followed key principles of working with Aboriginal and Torres Strait Islander peoples, including being based on relationships and privileging Aboriginal leadership (23, 27). Relationship building forms an integral part of how Aboriginal and Torres Strait Islander people prefer to interact with health professionals and if based on mutual respect then the communication and conversation will be fruitful (23). Regarding Aboriginal leadership, the research team included culturally competent, experienced and confident Aboriginal researchers and support workers to assist and support the delivery of the program. The team leaders included senior Aboriginal researchers from WA (MK) and NSW (JC) who provided oversight and monitoring of the research. The senior Aboriginal researcher from WA (MK) also provided mentoring to the primary researcher (MJRG) who was a senior Noongar Wadjuk woman. A senior WA researcher on the team (AMH), who was not Aboriginal, worked closely with both WA Aboriginal researchers to take a stance of critical reflexivity that included letting go of certainties and working outside of one's own comfort zone (28, 29). It involves shared learning and building a respectful relationship between Aboriginal researchers and non-Aboriginal team members. This stance recognized privileging Aboriginal ways of working within an Aboriginal and Torres Strait Islander ethics framework, and the importance of Aboriginal and Torres Strait Islander values and principles being central to the research. Both MK and AMH visited both groups at regular intervals to work with Elders and staff.

Aboriginal Elders living on Noongar Boodjar (Country) have their own cultural practices and dialect although they engage in similar ways it is easy to make the mistake that they will work the same way, but this “one size fits all” approach does not work. One Group accepted male participants, the other did not. One group included a welcome to Country, the other did not. The PA program ran according to the needs and expectations of the Elders who would at times correct the Aboriginal and non-Aboriginal staff on how they needed to meet and work together, while the lead Researcher (MJRG) maintained a neutral position, that allowed for the voices and direction from the Elders collaborating with the Aboriginal health workers to develop their own terms (rules) for working successfully together. We were always mindful of not overstepping our boundaries as visiting researchers. This method of working with the Aboriginal Elders on Noongar Boodjar was in accordance with their “ways of working,” where could reinforce how they wanted the PA groups to operate. These relationships form part of Indigenous methodology that is essential to successfully deliver programs when working with Aboriginal and Torres Strait Islander peoples (30). The research was undertaken between February 2019 and July 2021. Ways of working commenced with building relationships, leadership from Aboriginal researchers and community members and continued through to operating the program each week in a way that is comfortable and safe for each person that attended the groups (31).

#### Program Interruption

After 12 months of operation there was a gap in program delivery of 6 months in 2020 due to the COVID-19 pandemic restrictions. The program resumed at 18 months and continued for another 6 months.

### Ethics

Ethics approvals for the study were obtained from WA Aboriginal Health Ethics Committee (HE 842) and Curtin University (HE number 2018-0425). All participants provided written, informed consent prior to participating in the study.

### Participants and Setting

Aboriginal Elders who resided on Noongar Boodjar in the South West of WA were invited to participate. Inclusion criteria were being aged 45 years or older, able to attend the group and participate in exercises. Participants were Noongar Elders and Elders who lived on Noongar Country but came from other Boodjars (Countries) in WA and other regions in Australia. Groups operated at local community centers. Participants were asked to obtain a medical clearance to attend the program. Two groups of Elders were recruited, one from a regional area and one from a metropolitan area. The metropolitan group collaborated with a government department and the regional group was conducted by the local Aboriginal Controlled Community Health Service (ACCHS). The government department had an Aboriginal program director and provided an Aboriginal project officer to support the metropolitan group. Both groups were supported by Aboriginal health workers and non-Aboriginal health workers (physiotherapist and exercise physiologist, program assistants, research assistants) as an operational team. Non-Aboriginal health workers who had undertaken previous work with the Aboriginal Elders were recruited to the team. These workers were guided by their own cultural competency training and worked closely with the Aboriginal team members, so they were mindful of awareness of ways of working with Aboriginal Elders.

The program was operated in local community centers which were culturally safe spaces. These centers were used by other local Aboriginal people for their cultural activities and multi-cultural activities that the local Aboriginal Community participated in, so they felt comfortable in using these venues. Both venues were open and welcoming of the program, and offered use of suitable rooms. There was a regular traffic of Aboriginal faces coming in and out of both community centers. The project officer at the metropolitan group and the ACCHO in the regional setting, both in consultation with the Elders, discussed the venue and Elders provided confirmation that the venue was suitable after attending for several weeks.

### Intervention

Ironbark means standing tall and strong like the Ironbark tree (32). The Ironbark program in NSW was named this as part of developing the program. After discussion, this name was retained for the project in WA, because WA has the same Ironbark tree with different colored flowers. The Ironbark was a weekly program that included PA and yarning, along with morning tea. The research team adapted the original material from NSW Ironbark program to suit the preferences of Aboriginal Elders residing on Noongar Country and referenced a holistic, decolonizing approach to implement it. This decolonizing approach was about understanding the challenges and complexities of working with Aboriginal Elders on Noongar Country and recognizing it is a positive strategy toward providing better supportive and stronger research practices that will benefit both the community and the researcher. Too often in the past research was done without the informing, the approval and consent of Aboriginal people and evaluations resulted in recommendations and decisions made about Aboriginal people by non-Aboriginal people which almost always failed (4). Decolonization is also about understanding the relational aspects of communities that are important, respectfully understanding the diversity that exists within communities and groups, and always collaborating with them, enabling their voices to be heard, particularly in our research, the decision-making aspects of the weekly PA program (29, 33). Aboriginal health workers at both sites endeavored to make the PA program as comfortable as possible for the Elders by providing group and individual support for all aspects of attendance and participation. They worked with each Elder to ensure that they were comfortable doing the exercises and this continued until they were confident to do them. The program focused on exercises that have been shown to be effective in reducing falls, namely exercises that have a strong balance and functional component (34). This 1-h exercise component was delivered alongside yarning circles (35). Yarning is a respectful and culturally acceptable way to engage with Elders, for it helps to improve and build lasting relationships simply by honoring the Elders through actively listening (36). The exercises were led by the health professionals who provided the exercise training supported by Aboriginal health workers, in a space that was culturally safe and secure. Each session commenced with a warm-up, then included a variety of lifting light weights, ball activities, balance and strength exercises, as well as walking and occasional dancing (in modern, casual style) to music. Individual advice regarding exercise was provided as appropriate for participants by the attending health professional. A home exercise program (HEP) was prescribed based on the exercises done in the group. Participants were given handouts of the exercises and encouraged to complete their HEP twice weekly. Health educational topics relevant to fall prevention were discussed in the yarning circle using topics in the Ironbark manual (37). In the WA translation of the Ironbark program, participating Elders assisted to plan the schedule for yarning sessions and chose additional topics they felt were relevant to their needs, including those that focused on managing their health. Asthma, diabetes, heart disease and cancer were examples of popular topics for discussions. Yarning circles were facilitated each week by the Aboriginal project officer or Aboriginal health workers.

### Outcomes

Health outcomes evaluated were:

(i) Physical function - measured using the Short Physical Performance Battery (SPPB) (38). This is a hierarchical test of standing balance, participants' usual gait speed, and lower limb strength (standing five times from a seated position in a chair). Each test is scored on a 0–4 scale and summed for an overall score range of 0–12, with zero indicating the lowest physical performance, and 12 indicating the highest performance. Gait speed and lower limb strength were also evaluated as individual measures since both are measures of independent physical function in older adults (39).

(ii) Functional mobility - measured using the timed up and go (TUG) test that measures the time it takes in seconds for a person to stand from a chair and walk three meters, turn around, return to the chair, and sit down (40).

(iii) Cardiovascular health–measured by waist circumference (cm) which is an easy anthropometric measure that predicts cardiovascular disease (41). Weight (kgs) was also measured since exercise is known to be an effective means of reducing weight (42), which can in turn improve cardiovascular risk profiles (43).

(iv) Falls self-efficacy–measured participants' concern about falling using the Short Falls Efficacy Scale International (Short FES-1) (44), where a minimum score of 7 indicates no concern about falling, and a maximum score of 28 indicates severe concern. Since the Ironbark was designed as a fall prevention program it was felt that participants' concern about falling might be impacted by the program.

(v) Health related quality of life (HRQoL)–measured using the Assessment of Quality of Life (AQoL-4) instrument (score range 12–48, lower score indicates better HRQoL) (45). This instrument has been validated and found to be reliable in Australian populations and has been tested in an Aboriginal and Torres Strait Islander population (46).

Participants' feedback about the program was undertaken using a questionnaire and “yarning” to capture the cultural appropriateness of the PA program and how it could be improved. These types of feedback had previously been used by researchers to evaluate the pilot trial that was conducted in NSW (10). The questionnaire contained closed-ended and open-ended items. It aimed to seek participants' feedback about whether they found the program relevant, useful, and culturally appropriate and gather suggestions for any changes to the program. The feedback provided by participants through yarning about the PA program has been reported separately (26).

Demographic data gathered at baseline included age gender, language, education, number of health conditions, history of falls (defined as “an event which results in a person coming to rest inadvertently on the ground or floor or other lower level”) (47), exercise in the past 12 months, and number of prescribed medications taken. The number of medications was considered a feasible measure to collect as a surrogate marker of chronic disease (48).

### Procedure

Aspects of the procedure have been described previously (26). Briefly, the development of the research procedure was underpinned by the guidelines for ethical conduct for conducting research in Aboriginal and Torres Strait Islander Peoples and Communities (49). Consultation with WA Aboriginal communities occurred prior to program commencement. Initially researchers from NSW met in WA with Aboriginal led organizations and interested researchers, including senior Aboriginal researchers (MK and JC) from both NSW and WA. This was undertaken through local Aboriginal organizations in Perth and a number of Aboriginal Controlled Community Health Organizations (ACCHO) in regional areas. These organizations include local Elders and, more broadly, community representation. The success of the Ironbark program in NSW and interest in having a similar program for Elders in WA was discussed and both groups sought more feedback from their communities. Meetings were flexible in time and frequency depending on questions that arose and communication occurred throughout these preparation phases. This included the senior WA researchers (one Aboriginal–MK, one non-Aboriginal–AMH) meeting with community Elders by meetings arranged through the two interested organizations. After ~2 years of formal and informal consultations it was agreed to form two groups that would evaluate the program. The final team, including one ACCHO, one WA government department that led Aboriginal public health strategy, the original NSW Ironbark researchers and WA researchers was formed and AMH and MK led applications for funding for the research. When the program was operationalized each Elder's group developed their own terms of reference. This described their values and expectations about how the program would be conducted to meet the needs of the community (21). Understanding that one's own cultural beliefs, values, attitudes and practices may vary considerably and being able to accept and be respectful of these differences was fundamental (18, 50). Having knowledge of the history of older Aboriginal and Torres Strait Islander peoples which includes trauma, loss and illness, means that steps can be taken toward mutual understanding and building partnerships for more respectful and meaningful communication, leading to successful outcomes (3, 4).

Health outcomes were measured at baseline, six, and 12 months and a final assessment was completed at 24 months at the conclusion of the program. Outcome measurement days were led by the Aboriginal student researcher, who was experienced at leading data collection in communities, with the physical outcome assessments being supervised by the lead physiotherapist researcher. Participants were administered the feedback questionnaire at the 12 months timepoint, or if they were not available at the 12-month timepoint they were asked to answer the questionnaire at the 24-month timepoint. Data collection was supported by the Aboriginal health workers, the Aboriginal project officer and Aboriginal research assistants. Training for health workers and research team assistants to assist with the data collection was provided prior to program commencement and at intervals throughout the program. The Ironbark research team from NSW and WA completed training together prior to the PA program commencement in the South West of WA. This combined team was made up of an Aboriginal researcher (JC) who was the former advisor of the original Ironbark training, the WA Aboriginal lead researcher (MJRG) and the WA Aboriginal project officer and Aboriginal project officers and health workers who would be present at both Elders' groups during the 2-year PA program.

### Statistical Analysis

Analyses were completed using STATA version 17.1 (StataCorp. 2019, Stata Statistical Software: Release 17. College Station, TX: StataCorp LLC). All data from both regional and metropolitan groups were combined for analysis. All health outcomes data were summarized using descriptive statistics and presented using frequency distributions for categorical data and means and standard deviations or medians and interquartile ranges (IQR) for continuous data. Linear mixed models and mixed effects negative binomial models, with random subject effects, were used to examine longitudinal continuous and count outcomes over four timepoints (baseline, six, 12 and 24 months). Model results were summarized using marginal mean estimates and 95% confidence intervals. As mixed effects models use maximum likelihood estimation to estimate parameters based on assumed probability distributions, all available data points, regardless of missing timepoints, were included in analysis. Data obtained from the feedback questionnaire were summarized using descriptive statistics. Data from open-ended questions were coded and summarized using frequency and percentages.

#### Sample Size

For a within group repeated measures ANOVA over four timepoints a sample of *n* = 12 has 90% power to detect an effect size f = 0.41. Feedback from the community partners suggested that it would be reasonable to expect each group to consist of between 7 and 10 regular participants.

## Results

### Program Delivery

There were 46 sessions delivered in year one of the program and 25 sessions in year two at the metropolitan site (36/31 in the regional site). Sessions were timed to fit into school terms with some gaps in school holidays and around public holidays. The reduction in sessions in year two was due to the COVID pandemic lockdown.

### Participant Flow Through the Study

**Figure 1:**
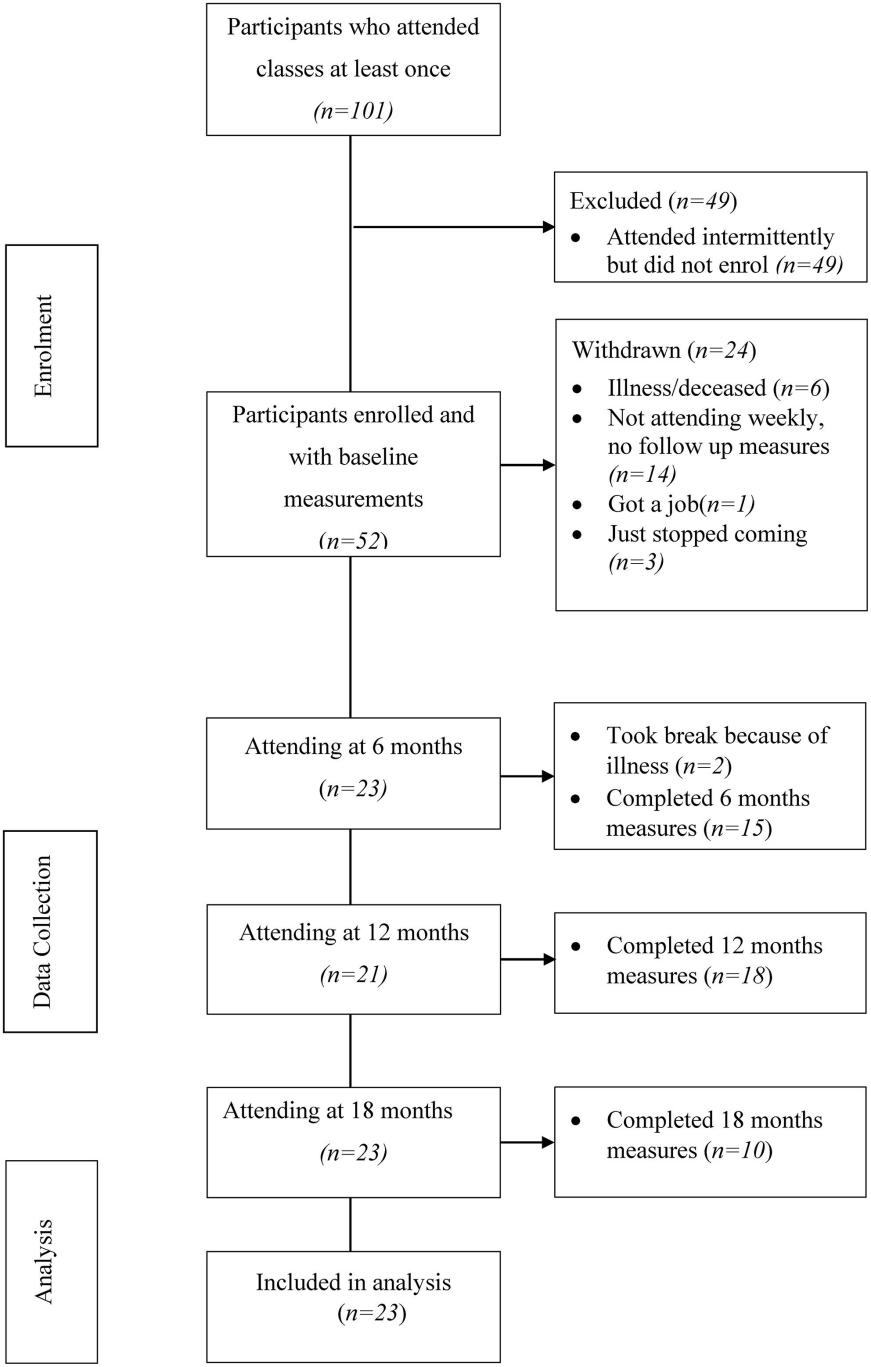
Participant flow through the study.

The flow of participants through the study is presented in Figure 1. There were 52 older Aboriginal people who enrolled and undertook baseline measurements, of these 14 (26.9%) did not attend the groups regularly, 6 participants withdrew due to medical illness or died, while 3 stopped attending with no reason stated. There were 23 participants who regularly attended the two groups. Fourteen (60%) participants attended at least 50% of all weekly sessions over the 2 years and 9 (40%) attended between 30 and 40% of sessions. All available participant data were included in analyses. Participants' characteristics are presented in Table 1. Participants had a mean age of 62 (±10.8) years and 21 (91.3%) participants were female. Participants took a median (interquartile range) of four (3–5) medications.

**Table 1:** Participants' characteristics (*n* = 23).

**Characteristic**	***N* (%)**
Site *n* (%)	
Regional	13 (56.52)
Metropolitan	10 (43.48)
Age, mean (SD), years	62.5 (10.85)
Gender, female, *n* (%)	21 (91.3%)
Language, *n* (%)	
English spoken as first language	22 (95.65)
Education, *n* (%)	
Primary school	4 (17.39)
Completed year 10	8 (34.78)
Completed year 12	4 (17.39)
Completed higher education	7 (30.43)
Falls history, *n* (%)	
Falls in the last 12 months	7 (30.43)
Injury from fall, *n* %	4 (57.14)
Attended an exercise class in last 12 months^a^, *n* %	5 (21.73)
Medications	
Number of medications, median (IQR)	4 (3–5)
More than four medications, *n* (%)	10 (43.47)
Primary medical conditions^b^, *n* (%)	
Respiratory condition	3 (13)
Diabetes	6 (26)
Musculoskeletal (low back pain, gout arthritis)	3 (13.04)
Cardiovascular disease (including hypertension)	12 (52.17)
Other medical conditions^c^	13 (56.52)

a *At least once per week in the last 3 months*.

b *Participants could have more than one health condition*.

c *Including cancer, renal disease, depression*.

### Health Outcomes Measurements

Predicted marginal means for physical function (SPPB, gait speed, lower limb strength), functional mobility (TUG test), cardiovascular health (waist circumference, weight), falls self-efficacy (Short FES-1) and HRQoL (AQOL-4D) at time points of six, 12 and 24 months compared to baseline are presented in Table 2.

**Table 2:** Predicted marginal mean health outcomes over four time points.

	**Margins**	**95% CI Lower**	**95% CI Upper**	*P*-value^*^
**Short physical performance battery (SPPB score range 0–12)^#^**				
Baseline	8.85	8.10	9.61	
6 m	9.55	8.69	10.41	0.110
12 m	10.28	9.44	11.13	<0.001
24 m	9.60	8.59	10.60	0.140
**Gait speed (meters/second)^&^**				
Baseline	0.81	0.69	0.93	
6 m	0.75	0.61	0.88	0.340
12 m	1.14	1.01	1.27	<0.001
24 m	1.11	0.95	1.26	<0.001
**Chair stand test (seconds)^$^**				
Baseline	17.13	14.93	19.33	
6 m	13.11	10.84	15.38	0.006
12 m	13.53	11.40	15.67	0.008
24 m	14.42	11.66	17.18	0.100
**Timed up and go test**, **(seconds)^$^**				
Baseline	11.62	9.40	13.84	
6 m	10.46	8.11	12.81	0.330
12 m	8.79	6.77	10.82	0.010
24 m	10.63	7.89	13.38	0.490
**Waist circumference (cm)**				
Baseline	111.36	102.93	119.79	
6 m	107.19	98.42	115.95	0.060
12 m	109.02	100.30	117.73	0.270
24 m	107.97	98.86	117.08	0.180
**Weight (kg)**				
Baseline	82.94	73.12	92.75	
6 m	81.17	71.11	91.23	0.320
12 m	82.24	72.33	92.15	0.640
24 m	80.65	70.49	90.81	0.230
**Falls self-efficacy (FES-1, score range 7–28)^±^**				
Baseline (ref)	8.77	6.87	10.67	
6 m	10.81	8.42	13.21	0.170
12 m	11.89	9.84	13.93	0.020
24 m	7.90	4.65	11.14	0.640
**Health-related quality of life, (AQOL-4D score range 12–48)^€^**				
Baseline	16.48	14.57	18.38	
6 m	16.94	14.83	19.05	0.580
12 m	17.95	15.98	19.93	0.030
24 m	17.24	14.68	19.79	0.480

* *Mean comparison from baseline;*

# *SPPB score range 0–12, higher score indicates better mobility;*

& *faster speed indicates better mobility;*

$ *faster time indicates better mobility;*

± *FES-1 score range 7–28, low score indicates no concern, maximum score of 28 indicates severe concern about falling;*

€ *AQOL-4D score range 12–48, lower score indicates better HRQoL*.

#### Physical Function

**Figure 2:**
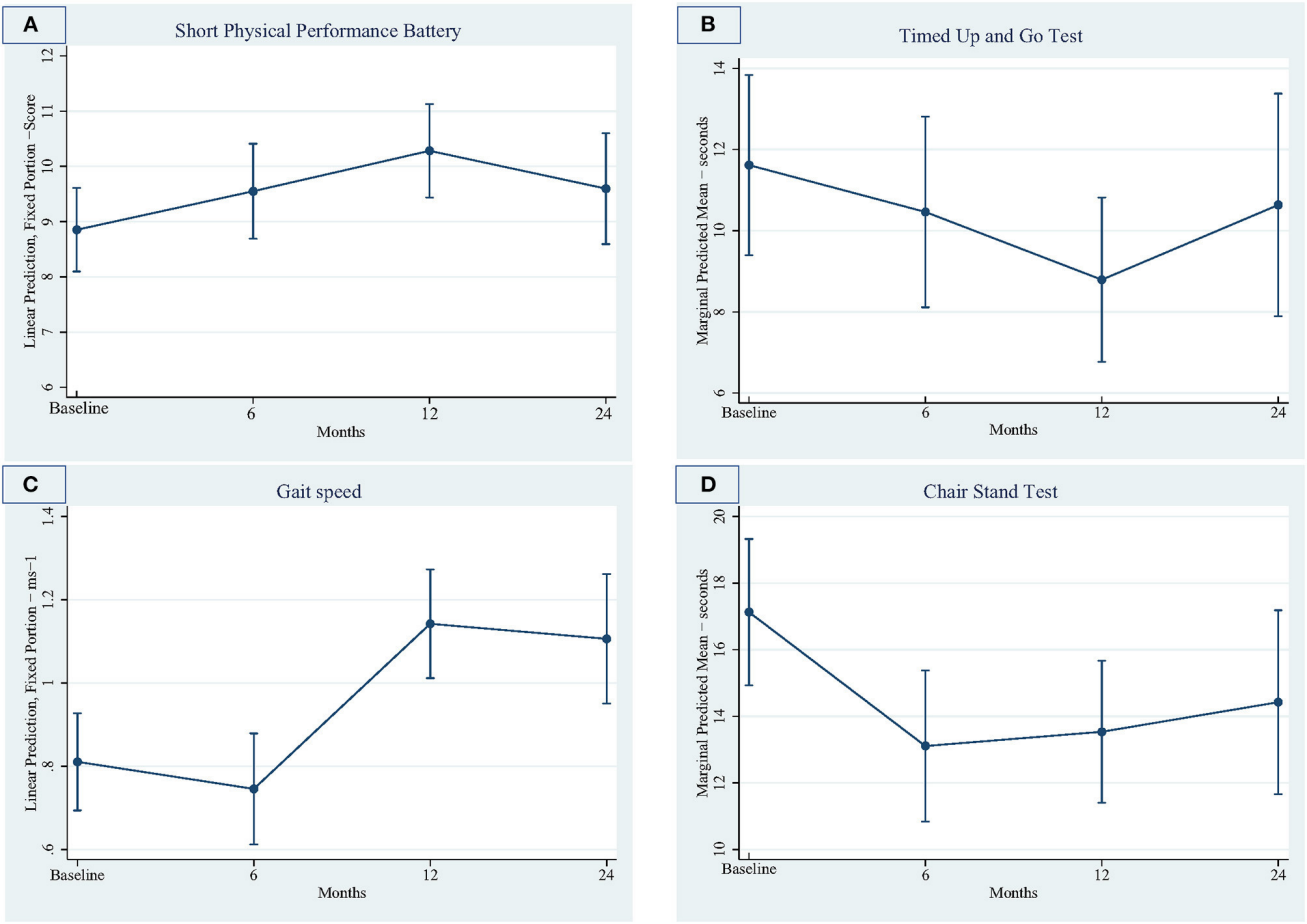
Physical function outcomes over four timepoints. **(A)** Short physical performance battery, **(B)** Timed up and go test, **(C)** Gait speed, **(D)** Chair stand test.

Physical function measures are summarized in Figure 2, with comparisons between baseline and follow-up periods presented in Table 2. Physical function (as measured by the SPPB) significantly improved at 12 months compared to baseline. Scores were also improved at 24 months compared to baseline, but the improvement was not significant. Gait speed showed significant improvement at 12 and 24 months compared to baseline. Lower limb strength (chair stand test) was significantly improved at 6 and 12 months compared to baseline. Functional mobility showed significant improvement at 12months compared to baseline.

#### Cardiovascular Risk Factors

**Figure 3:**
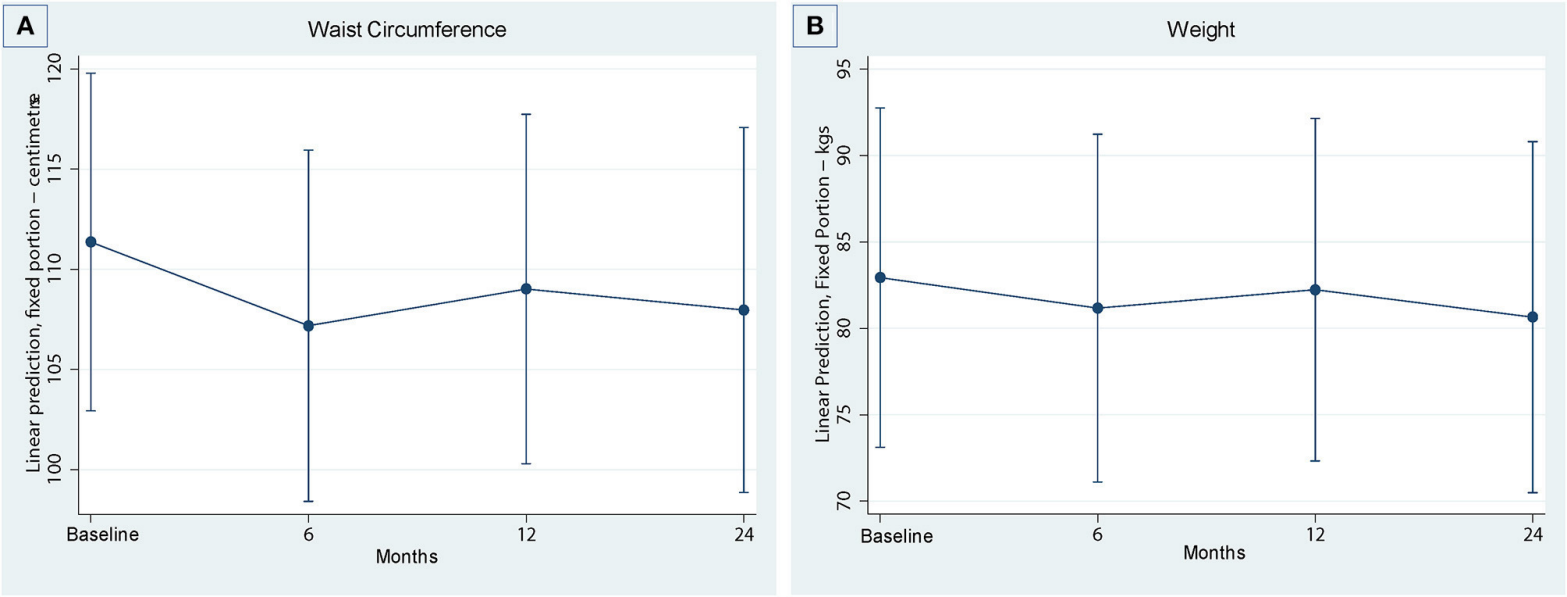
Cardiovascular outcomes over four timepoints. **(A)** Waistcircumference, **(B)** Weight.

Cardiovascular risk factors are summarized in Figure 3, with comparisons between baseline and follow-up periods presented in Table 2. There were improvements in cardiovascular risk factors (waist circumference and weight) at 6, 12 and 24 months compared to baseline, but these changes were not significant.

#### Falls Self-Efficacy and Health-Related Quality of Life

**Figure 4:**
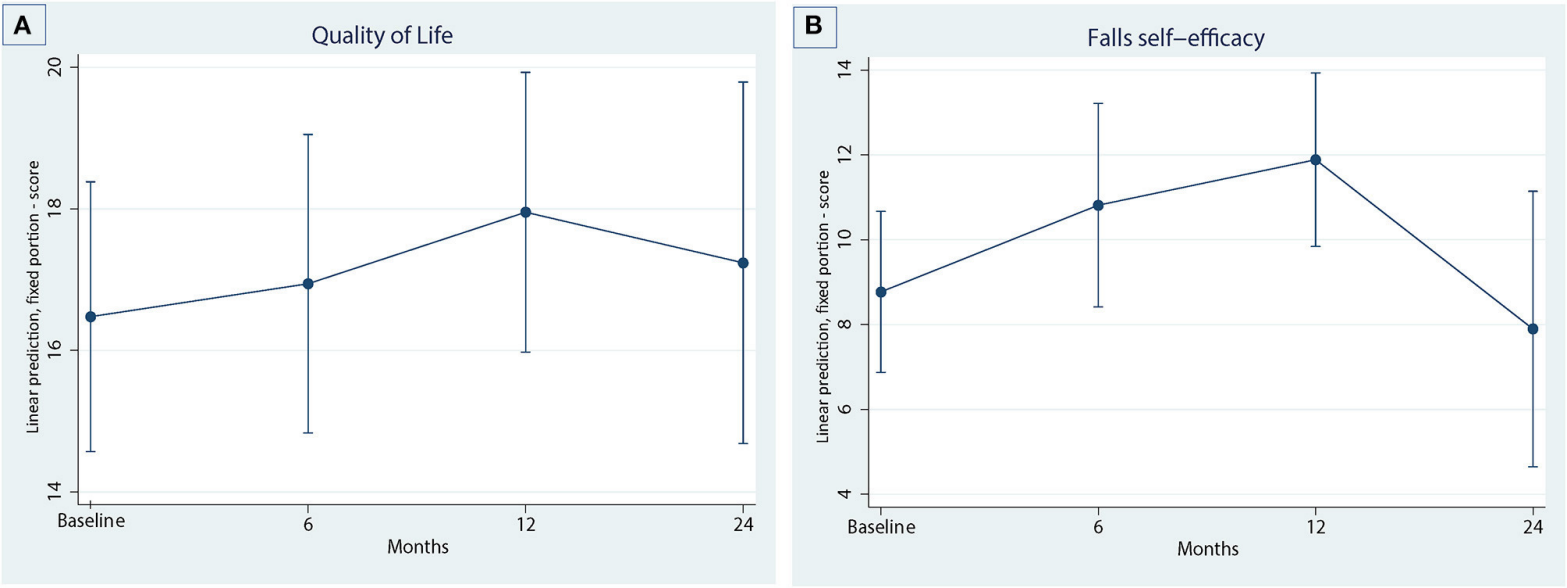
Quality of life outcomes over four timepoints. **(A)** Quality of life, **(B)** Falls self-efficacy.

Falls self-efficacy and HRQoL are summarized in Figure 4 with comparisons between baseline and follow-up periods presented in Table 2. Falls self-efficacy significantly declined at 12 months compared to baseline. Further analysis of this outcome was conducted by using linear mixed models with random subject effects to examine FES scores at 24 months compared to 12 months as compared to baseline. Results demonstrated that falls self- efficacy scores at 24 months significantly improved compared to 12 months [mean score at 24 months: 7.90 (95% CI 4.65, 11.14) compared to 12 months: 11.89 (95% CI 9.84, 13.93): *p* = 0.03], with participants' scores returning to less than baseline levels. HRQoL was significantly declined at 12 months compared to baseline, but not at 24 months.

### Participants' Feedback Regarding the PA Program

Participant feedback regarding the program is presented in Table 3. Eighteen participants provided feedback at the 12-month timepoint and five at 24 months. All participants enjoyed the program and agreed that it was culturally appropriate. Some participants [*n* = 14, (82.6%)] were willing to pay a fee to continue engaging in the program, while four participants were not. Most participants suggested they would be willing to pay a fee of $5, with four participants suggesting one dollar and one participant suggesting $10. Almost all participants suggested at least one improvement that could be enacted in the program. Improvements that were suggested were more outdoor activity [*n* = 12, (52.2%)], more exercises (*n* = 6), a different venue (*n* = 5) and a larger group (*n* = 4). Participants who reported improvements in their health (*n* = 19, (82.6%)] reported improved balance, confidence and a renewed desire to walk more, particularly in public places. Some participants (*n* = 7) responded that they found it difficult to complete their home program. Reasons for not completing home program were: not liking to walk alone, not liking walking at all, lack of motivation or doing other exercises outside of home and family commitments.

**Table 3:** Participants' feedback about the program (*n* = 23).

**No**	**Question**	**Response**	***n* (%)**
1	Is the program relevant to your needs?	Very relevant	18 (78.26)
		Somewhat relevant	5 (21.74)
2	Was the group discussion useful?	Very useful	17 (73.92)
		Somewhat useful	6 (26.08)
3	How much did you know about falls prevention before the program?	Had some idea	11 (47.83)
		Already knew a lot	5 (21.74)
		Not much or nothing at all	7 (30.43)
4	How much do you know about falls prevention now the program has finished?	A lot	22 (95.65)
		Nothing at all	1 (4.35)
5	Was the venue suitable?	Very suitable	21 (91.30)
		Somewhat / not at all	2 (8.70)
6	Was the program culturally appropriate?	Yes	22 (95.65)
		Somewhat	1 (4.35)
7	Did you have time to come to every session?	Yes	20 (86.95)
		No	3 (13.05)
8	Would you like to continue being involved in the program?^#^	Yes	22 (95.65)
9^*^	Would you be willing to pay a small fee to keep the program running?^&^	Yes	19 (82.60)
		Not sure	2 (8.69)
10	Was transport to the program a problem for you at any stage? ^&^	No	20 (86.95)
		Yes	1 (4.34)
11	Has this program improved your confidence with walking?^#^	Yes	20 (86.95)
		Unsure	2 (8.69)
12^*^	Do you feel your health has improved since attending the program?^#^	Yes	19 (82.60)
		No	1 (4.34)
		Not sure	2 (8.69)
13^*^	Were you able to complete your home exercises? ^&^	Yes	16 (69.56)
		No	5 (21.73)
14	Would you recommend this program to others?^#^	Yes	22 (95.65)

* *Participants could also provide an open-ended response to this item;*

# *Missing data n = 1;*

& *Missing data n = 2*.

## Discussion

Aboriginal Elders on Noongar Boodjar (Country) who participated in a 2-year PA program made significant improvements in physical function. Participants showed significant improvement in gait speed, lower limb strength and functional mobility at 12 months. These changes were reflected in the improvement in the SPPB (a composite measure of physical ability) with scores increasing from 8.8 at baseline to 10.28 at 12 months. Since a score lower than 10 is predictive of all-cause mortality, these changes demonstrate how the program was of positive benefit to participants' health (51). Mean gait speed significantly improved up to 24 months (1.11 m/s) compared to baseline (0.81 m/s) showing the program led to some sustained physical improvements. Baseline gait speed was below normative values for age-matched older adults' community walking speed but speed at both 12 and 24 months aligned with normative community values (52). Gait speed is predictive of functional ability and general wellbeing (53). In the delivery of the NSW Ironbark program gait speed improved to 0.94 m/s (10), however the NSW program ran for 6 months whereas our program continued for 24 months. Our findings suggest that with resources to run the program for longer, older Aboriginal and Torres Strait Islander adults may make even greater improvement. There was some decrease in physical function overall at 24 months compared to 12 months including the SPPB, TUG test and chair stand test. This corresponded with the closure of the program during the COVID 19 pandemic. However, after participants returned to the program improvement was still observable at 24 months compared to baseline in most measures. Our results concur with other studies that have evaluated PA programs for older Indigenous peoples (10, 11, 14, 54).

Cardiovascular risk factors (both waist circumference and weight) improved throughout the trial, even after the gap in program delivery in the second year, although not significantly. In the NSW Ironbark program participants showed a significant improvement in BMI. The WA program ran over 2 years and there were some sessions on nutrition, similar to the NSW program (10). Our program may have benefited from additional nutrition-focused interventions to increase impact on cardiovascular risk factors, since diet is known to impact on cardiovascular health (42). We also found that HRQoL showed small but significant decline at 12 months compared to baseline, and while improved at was still declined at 24 months compared to below baseline measures. This could have been due to the COVID pandemic as the social restrictions and class closure occurred at around the 12- month to 18-month time period of the program. COVID pandemic impacts on communities are known to have caused adverse effects on mental health for populations internationally (55).

### Elders' Feedback and Program Translation

Elders' feedback regarding the program was highly positive. Participants stated the program was excellent and culturally appropriate and they reported feelings of confidence that developed from the program. This was important as Aboriginal and Torres Strait Islander people are not likely to attend health services that are not provided in a culturally appropriate manner (18, 56). Previous research has found that Indigenous peoples know what their health burdens are and there needs to be a partnership approach to addressing existing barriers that prevent Indigenous participation in PA programs (18, 25, 56). Researchers should seek to actively listen to the voices of Indigenous people when building meaningful, culturally appropriate service provision and delivery for Indigenous populations (18, 25). PA programs should be designed using an Indigenous perspective, that ensures a decolonizing approach is taken. This will include elements of appropriate engagement, building leadership qualities, giving and receiving respect and providing a safe place for “yarning” as part of its conception. These qualities help improve communication and address the social determinants of health for disadvantaged people and communities, making the program more likely to succeed (18, 21, 56). Planning programs that take account of cultural identities is fundamental to ongoing program success (57, 58). Our findings are supported by other research that has found that PA programs designed with a decolonizing approach have successful outcomes among Indigenous peoples (11, 12, 18, 19). We noted the Elders commented on cost, and future translation needs to continue to address subsidizing the cost of PA programs. Effects of colonization have caused ongoing negative effects on social determinants of health, including education, employment and access to health services (3). A recent Australian national report summarizes that social determinants of health strongly impact on Aboriginal and Torres Strait Islander peoples' health. Aboriginal and Torres Strait Islanders who are more advantaged across social and economic measure have better health (59).

The success of the translation of the Ironbark program was that it was developed with the fundamentals of Aboriginal research methodology (24, 30). This research is the first to our knowledge to translate a culturally appropriate PA program for Aboriginal peoples in the South West of WA. Our PA program (the Ironbark program) was able to be successfully tailored for older Aboriginal people in WA because it took a working together approach, which included recognizing cultural identities. In order to provide better solutions for Indigenous communities, there needs to be an incorporation of “ways of working” from an Indigenous perspective that acknowledges language and customs and provides strong development of a culturally safe space (2, 4).

### Strengths and Limitations of the Research

Strengths of the program were the culturally appropriate design which led to Elders attending regularly, with feedback showing that 22 of the 23 Elders thought it was culturally appropriate and wanted to keep attending the program. The physical outcomes were measured at regular timepoints by trained Aboriginal health workers assisted by health professionals, using validated and reliable measures of physical function (38). Hence evidence for physical improvement was robust. A major strength of the study is to the authors' knowledge it is the first study to evaluate the effect of a culturally appropriate PA intervention on the physical function of Aboriginal Elders living on Noongar Boodjar (Country).

A major strength of the Ironbark PA program in the South West of WA was that the majority of the program team were Aboriginal people who partnered with non-Aboriginal team members to implement and facilitate the exercise and yarning circles. The program was a unique opportunity that enabled older Aboriginal people to take part in PA, because it was culturally acceptable. We delivered the program using an Indigenous “ways of working” approach, that was relevant to the two communities. Maintaining strong relationships throughout the research was fundamental to its success. The research was only undertaken after an extensive 2-year community consultation that enabled Aboriginal and non-Aboriginal researchers to work closely together in a manner that privileged Aboriginal people's worldviews (27). Extensive consultation with Aboriginal community groups is an important requisite to being able to honor ways of being, knowing and doing (56).

There was a major disruption to program delivery due to the COVID 19 pandemic in 2020. The main social restrictions period of over 4 months prevented program delivery, with subsequent delay in recommencement due to new program requirements that needed to be operationalized. The research team provided support by telephone and text messages, with the aim of providing mental health support and encouraging Elders to continue their home exercises and managing their health. Elders provided Facebook support to each other through these difficult times, which was felt to contribute to participants resuming regular attendance when the program recommenced. A limitation of the research was that we did not collect data that measured the amount of home exercise that participants completed. Another limitation was that we did not re-assess participants when they returned to the program at 18 months after a 6 month pause in the program. Requirements related to COVID restrictions were perceived as causing significant increased procedural burden to both staff and participants and it was felt important to recommence the program with as few barriers as possible. To avoid participants and staff burden in future programs, it could be useful to undertake a single, simple weekly assessment that rates improvement, rather than multiple assessments. Elders who enrolled were always not able to attend weekly due to medical appointments, family commitments or community relationships. More flexibility in the program delivery might assist with these barriers. Our study was limited to providing one class per week. Additionally, not all Elders attended the program and it could be because they were not favorably disposed to the program. Also, not all Elders chose to enroll in the study or provide feedback about the Ironbark program even though they intermittently attended and participated. It would be valuable for future translation of the program, to gain these Elders' perspectives about how the program could be improved or tailored to increase regular attendance.

The research did not use an experimental design. Since the benefits of exercise for older adults are well-established (5). The Ironbark project in WA, of which this study formed a component, took an implementation science approach which asks how can established evidence be translated into clinical practice (60). No control group is a limitation as Elders may have made improvements without attending the program, although very few of the Elders reported engaging in other PA programs prior to or during our program. Elders provided valuable perspectives on the Ironbark program. It is also important to consider the views of the communities and key stakeholders about what they saw and felt was relevant to the success of the program (26, 56, 61). A future study to explore these perspectives is planned.

## Conclusion

Few PA programs are designed specifically for older Aboriginal and Torres Strait Islander peoples. Westernized health care delivery has not addressed and acknowledged the impacts of colonization which has created exclusion, and exposure to inequalities of health service delivery (2–4). Translating the Ironbark PA Program into the South West of WA with two older Aboriginal communities was successful because there was Aboriginal leadership and strong relationship building. The program provided a unique opportunity not previously available for older Aboriginal people. Participants demonstrated positive changes in their health and wellbeing, including significant improvements in physical function. The groups' success highlighted that having a culturally appropriate PA program that is flexible and designed with a decolonizing approach can attract and retain older Aboriginal people who are seeking to improve their health and wellbeing. The learnings from translating this PA program could be of assistance to other communities and researchers who are seeking to promote PA with older Aboriginal and Torres Strait Islander peoples. The Ironbark PA program was a positive step toward breaking down barriers and building strategies toward closing the gap in health disparities for Indigenous peoples worldwide. Encouraging leadership by the Elders throughout the program was part of the decolonizing approach of the program that led to its success. Further research that partners with Aboriginal and Torres Strait Islanders Elders and communities to expand culturally appropriate PA programs is required.

## Terminology

*Acknowledgment of Boodjar (Country):* Authors acknowledge that this research was conducted on Noongar Country. We respectfully acknowledge the Noongar peoples as the Traditional Owners of Country in the South West of Western Australia (WA), where this research has been conducted. We recognize Indigenous peoples of all Nations' continuing connection to lands, waters, and communities. We pay our respects to all Indigenous Elders past and present. Authors respectfully acknowledge all the Aboriginal and Torres Strait Islander communities and Nations who contributed knowledge to this research.

*Terms used by the authors*: When describing the setting and interviews authors have chosen the words Noongar, acknowledging that there are many different Boodjars of Noongar people in the South West of WA. The term Aboriginal Elders is used when discussing all participants, as the groups included Aboriginal Elders from more than one Boodjars in WA, as well as Nations in other States of Australia. Elders is used as a term for all participants, respecting their knowledge and age in the community. When discussing studies from other countries the authors have respectfully used the authors' descriptions of the participants and their Nations. When discussing Australian studies in general from around Australia, the term Aboriginal and Torres Strait Islanders is used. When discussing research methodology in general and Indigenous peoples worldwide, the term Indigenous is used.

## Data Availability Statement

The raw data supporting the conclusions of this article will be made available by the authors, without undue reservation.

## Ethics Statement

Ethics approvals for the study were obtained from WA Aboriginal Health Ethics Committee (HE 842) and Curtin University (HE number 2018-0425). All participants provided written, informed consent prior to participating in the study.

## Author Contributions

MJRG led the original drafting of the manuscript. A-MH led the study conception and design with MK, RI, JC, KH, MJRG, NB, VP, and AJ. MJRG led study procedures including ethics procedures, program delivery and data collection and management, with assistance from A-MH, TW, MK, VP, and NB. MJRG and A-MH completed statistical analyses with oversight and assistance from AJ. Revisions to the manuscript were addressed by A-MH and MJRG and approved by all authors for the final revised version of the manuscript.

## Funding

This study was funded by a Healthway Grant (Grant Number 31960). MJRG is a Noongar Wadjuk Ph.D. student supported by a stipend from the grant. A-MH was supported by a National Health and Medical Research Council of Australia Investigator (EL2) award.

## Conflict of Interest

NB was employed by South West Aboriginal Medical Service Aboriginal Corporation. The remaining authors declare that the research was conducted in the absence of any commercial or financial relationships that could be construed as a potential conflict of interest.

## Publisher's Note

All claims expressed in this article are solely those of the authors and do not necessarily represent those of their affiliated organizations, or those of the publisher, the editors and the reviewers. Any product that may be evaluated in this article, or claim that may be made by its manufacturer, is not guaranteed or endorsed by the publisher.
